# Study of the bone behavior around a neck preserving short stem implant: bone densitometric analysis over a span of two years

**DOI:** 10.1051/sicotj/2016025

**Published:** 2016-10-07

**Authors:** Tarek Abdel Shafy, Adel Sayed, Ahmed H. Abdelazeem

**Affiliations:** 1 Kasralainy Hospital, Faculty of Medicine, Cairo University Cairo Egypt; 2 Arab Contractors Hospital, Arab Contractors Medical Center Cairo Egypt

**Keywords:** MiniHip, Short stem, DEXA, Periprosthetic remodelling, Neck preserving

## Abstract

*Purpose*: Study the bone mineral density (BMD) changes and the remodelling process after implantation of a neck preserving short stem implant over a period of two years.

*Methods*: Using specific patients’ selection criterion, a prospective study was done including 26 patients. All were operated upon by a single surgeon using the MiniHipTM, (Corin, Cirencester, UK). Mean age was 42.5 years. Clinical and radiological evaluation was done. Periprosthetic bone density was measured by DEXA. First scan was obtained within 10 days after surgery and served as a baseline for comparison.

*Results*: The mean pre-operative Harris Hip score of 37.8 increased to 95.1 points two years post-operatively. BMD in the overall periprosthetic area showed a significant reduction during the first three months. Restoration to the original levels was reached in all zones except the most proximal zones at one year. A net increase was detected (+3%) after two years.

*Conclusion*: The neck preserving MiniHip short stem implant has proven to be a bone-friendly design. Significant bone remodeling process continues after the first year. Although bone resorption in the greater trochanteric region is still a problem, however, it has proven that the BMD in all the other periprosthetic regions including the calcar and the lesser trochanteric regions, are subjected to bone formation process over a period of two years.

## Introduction

The demographics of patients with hip disorders have changed over time. Patients are younger with high activity demands and have a longer life expectancy [1]. Periprosthetic bone resorption of the proximal femur is one of the main problems that affects the implants’ survival especially in young generations. To avoid this, implants that mimic the physiological load transfer are required. Load transfer depends on the implant geometry, size, and degree of stiffness, type and extent of coating, as well as implant stability and osseointegration to the host bone [2].

Recently, many different designs of short femoral stems have been introduced. These designs are focused on more proximal load transfer and a sound physiological behavior as well as reduction of the diaphyseal fixation [3]. Albanese et al. suggested that shortening the femoral stem might promote complete proximal load transmission and, consequently, greater physiological stress distribution [4]. A biomechanical study by Whiteside et al., in 1995, confirmed the advantage of preserving the neck of femur during hip replacement because the forces encountered during weight-bearing are transferred more homogenously to the proximal femur when the femoral neck is preserved. Furthermore, the study revealed the importance of the femoral neck in increasing the rotation stability of the implant [5].

According to this information, we started using one of the “partial collum designs” or “neck preserving” short stem implants [3] in our institution since 2009; MiniHipTM (Corin, Cirencester, UK) on a specific type of patient. We conducted this study, on a specific group of patients, aiming at studying the bone behavior or response to these implants through examining the changes in the bone mineral densities (BMD) in the first two years after implantation. Hypotheses were: (1) bone resorption occurs in the first three months, afterwards; the process of bone formation starts and reaches its maximum at one year, afterwards only minor changes will occur; (2) neck preservation will reduce the neck resorption process in the calcar and the lesser trochanteric regions.

## Patients and methods

Between August 2009 and May 2015, a prospective study was conducted on 26 patients who had a unilateral total hip replacement using a single short stem hip implant (ceramic on ceramic MiniHipTM, Corin, Cirencester, UK). The study was conducted to examine the bone behavior around this short stem. All patients were followed up clinically and radiologically for two years. The study was approved by the Institutional Review Board (IRB) of the Department of Trauma and Orthopaedics, Arab Contractors Hospital, Cairo.

The study was conducted on patients with special demographics excluding all patients with radiologically detectable osteopenia, abnormal deviation of the neck (anteversion > 25°, coxa valga > 150° or coxa vara < 115°), pathological fracture, previous metaphyseal osteotomies or deformities, and contralateral hip disease or replacement (Table 1). All surgeries were done using epidural anesthesia in a lateral position using the posterolateral approach and by the same surgeon (TS) in a single hospital.

**Table 1.** Patient’s demographics.

Number		26
Age	Range	21–50
	Mean	42.5
Sex	Males	20
	Females	6
Side	Right	15
	Left	11
Causes of replacement	Degenerative hip diseases	22
	Fracture neck femur – Garden IV	3
	Revision of loose resurfacing component	1
BMI (kg/m^2^)	Range	19–37
	Mean	27.3

### Clinical evaluation

Harris hip score (HHS) [6] was used for pre- and post-operative clinical evaluation for all patients at three months, six months, one year, and two years of follow-up.

### Radiological evaluation

Pre-operative radiographs were analyzed for femoral bone quality based on the Dorr classification [7]. The angle of the neck resection level was verified through templating; the mid-neck resection level was used. Femoral stem alignment was assessed on the immediate anteroposterior (AP) radiographs of the pelvis and classified as neutral, valgus, or varus. A neutral alignment was considered when the distal polished part of the stem was in contact with and parallel to the endosteum of the lateral cortex, and the upper medial part of the stem was in contact with the calcar area. A valgus alignment was considered when the stem was resting its distal polished part on the medial cortex. A varus alignment was considered when the stem was anchored by the tip to the lateral cortex, and its upper medial part was not in contact with the medial calcar area. To assess stability, length measurements from the superior tip of the greater trochanter to the distal tip of the implant were compared between immediate post-operative and long-term follow-up visits.

### Duel Energy X-ray Absorptiometry (DEXA)

**Figure 1.**
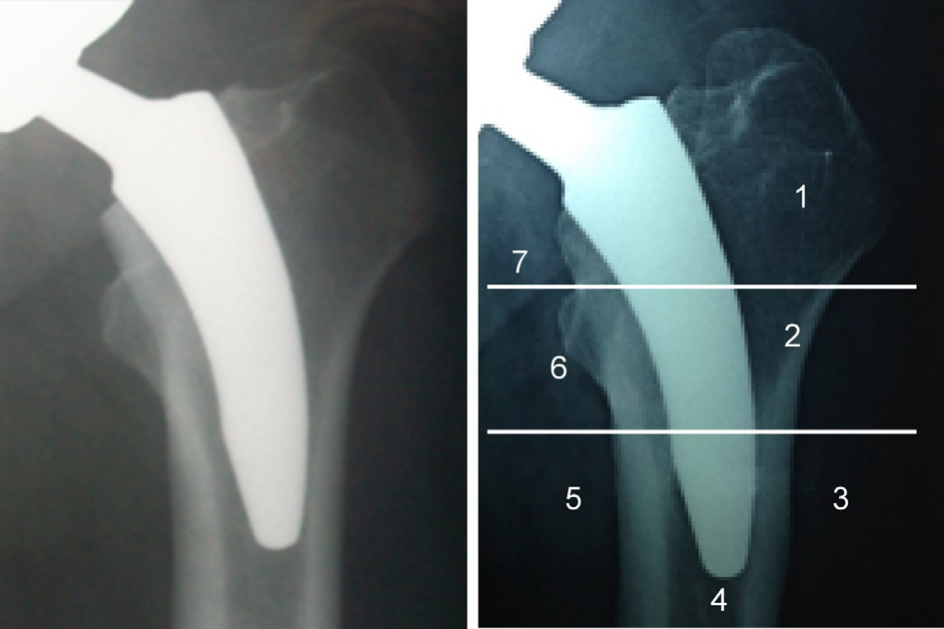
Regions of interest (RIOs) according to Gruen’s zones.

The periprosthetic bone density was measured by DEXA scan using specialized orthopedic software (*Lunar Prodigy Primo version PR* + *351125*). The scanning procedure, as well as the positioning of the patient and the degree of leg rotation, were standardized for all patients to guarantee high measurement accuracy. The DEXA scan was obtained within seven to 10 days after surgery and served as a baseline for the subsequent scans [8]. The proximal femur was divided into seven zones (Regions of Interest (ROIs)) according to Gruen et al [9]. The bone mineral content (g), area (cm^2^), and bone mineral density (BMD) (g/cm^2^) were calculated in the seven ROIs (Figure 1)*.*The BMD was measured laterally (Zones 1, 2, and 3) and medially (Zones 5, 6, and 7) around the stem and at least 1 cm distal to the tip of the stem (Zone 4). Zones 1–7 were combined to determine the total periprosthetic BMD.

Further scans were obtained at three months, six months, 12 months, and 24 months post-operatively. The post-operative change in BMD was assessed by comparing the subsequent BMD value with the BMD value measured immediately post-operatively. The difference was expressed as the percent change versus the baseline value.

### Statistical analysis

The statistical analysis was performed by a qualified biostatistician. Data were analyzed using the Statistical Program for Social Science (SPSS) version 18.0. Quantitative data were expressed as means ± standard deviation (SD). Qualitative data were expressed as frequencies and percentages. A probability (*p*-value) < 0.05 was considered significant, a *p*-value < 0.001 was considered highly significant, and a *p*-value > 0.05 was considered non-significant.

## Results

### Clinical results

The mean pre-operative HHS of 37.8 points (SD ± 14.9) increased to 95.1 points (SD ± 3.9) two years post-operatively (Table 2). In all cases, thigh pain was not observed at the six month follow-up evaluation. Highly significant differences between the pre-operative and the six month, one year, and two year HHSs using a paired sample *t*-test, which results in *p*-values < 0.001, were observed.

**Table 2.** Differences between the pre- and post-operative Harris hip scores (HHSs).

HHS	Mean	±SD	Mean Diff.	*p*-value
Pre	37.8	14.9		
After 6 months	83.1	12.4	−45.4	<0.001
After 1 year	92.7	3.3	−53.9	<0.001
After 2 years	95.1	3.7	−59.1	<0.001

Two complications were reported, which we think did not affect the results of our study. Acute Deep Venous Thrombosis (DVT) occurred in one male patient three weeks post-operation and a dislocation occurred in another case. That was a 48-year-old female patient who experienced a traumatic posterior dislocation after falling on the ground six months post-operatively. The dislocation was managed by closed reduction under general anesthesia and bed rest for six weeks.

### Radiological results

The immediate post-operative and subsequent radiographic findings are summarized in Table 3.

**Table 3.** Summary of immediate and post-operative follow-up radiographic results.

Radiographic parameter	Findings
Femoral morphology	
Dorr classification: *no. (%)*	Total no. = 26 hips (100%)
*Type A*	10 (38%)
*Type B*	16 (62%)
*Type C*	0
Femoral component alignment: *no. (%)*	Total no. = 26 hips (100%)
Neutral	25 (96%)
Varus	1 (4%)
Postop. limb length discrepancy mean and range *(cm)*	Mean = 0.8 cm
	Range = (0.5–1 cm)
Calcar atrophy: *no. (%)*	No. = 26 hips at 2 years postop.
Round-off	18 (70%)
Loss of calcar height	1 (4%)
Loss of calcar thickness	1 (4%)
Heterotrophic ossification:	No. = 26 hips at 2 year postop.
*No. (%)*	2 (8%)
*Grade I*	1 (4%)
*Grade II*	0
*Grade III*	1 (4%)
*Grade IV*	0
Stem osseointegration (stable): *no. (%)*	No. = 26 hips at 2 years postop.
*Bone bridging (spot welds) in at least one Gruen zone*	23 hips (88%)
* Cortical hypertrophy*	3 hips (12%)
Radiolucent lines: *no. (%)*	0
Stem tip reactive sclerotic line: *no. (%)*	1 (4%)

### Densitometry results

**Figure 2.**
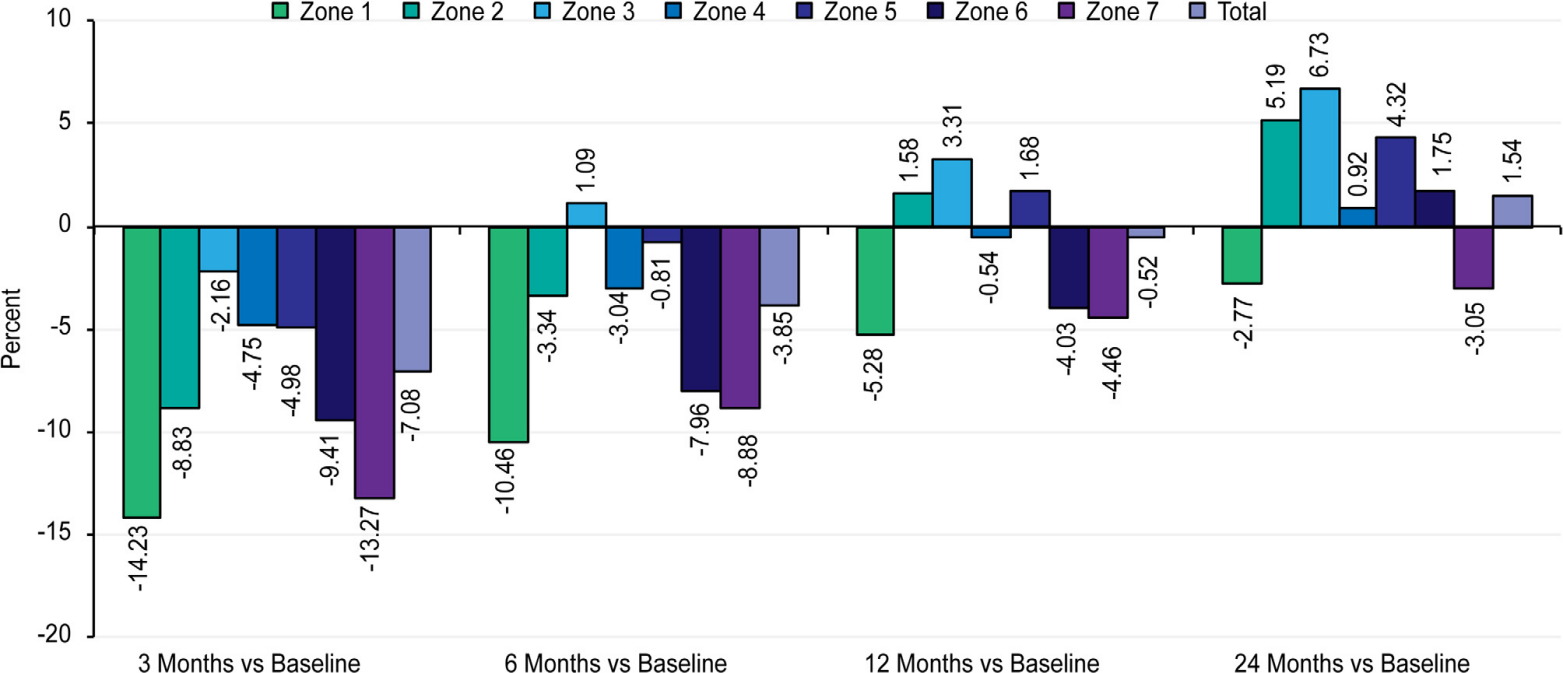
Bar chart showing the DEXA results 3, 6, 12, and 24 months as compared with baseline.

The mean results of the BMD in the seven ROIs were compared between immediate post-operative baseline values and subsequent follow-up results (Table 4 and Figure 2).

**Table 4.** The DEXA results 3, 6, 12, and 24 months compared with baseline.

Gruen zone	Immediate postop.(baseline value)		3 month postop. BMD		Percent change vs. baseline (%)	*p*-value	6 month postop. BMD		Percent change vs. baseline (%)	*p*-value	1 year postop. BMD		Percent change vs. baseline (%)	*p*-value	2 year postop. BMD		Percent change vs. baseline (%)	*p*-value
	Mean	SD	Mean	SD			Mean	SD			Mean	SD			Mean	SD		
Zone 1	0.73	0.20	0.63	0.21	−14.23	0.000	0.65	0.20	−10.46	0.000	0.69	0.19	−5.28	0.015	0.71	0.19	−2.77	0.074
Zone 2	0.99	0.17	0.90	0.19	−8.83	0.000	0.96	0.21	−3.34	0.005	1.00	0.21	1.58	0.217	1.04	0.20	5.19	0.000
Zone 3	1.78	0.22	1.74	0.23	−2.16	0.000	1.80	0.25	1.09	0.121	1.84	0.24	3.31	0.000	1.90	0.23	6.73	0.000
Zone 4	1.75	0.26	1.67	0.25	−4.75	0.000	1.69	0.24	−3.04	0.000	1.74	0.25	−0.54	0.330	1.76	0.27	0.62	0.000
Zone 5	1.76	0.29	1.67	0.27	−4.98	0.000	1.75	0.26	−0.81	0.351	1.79	0.26	1.68	0.258	1.84	0.27	4.32	0.002
Zone 6	1.52	0.39	1.37	0.37	−9.41	0.000	1.40	0.36	−7.96	0.000	1.46	0.36	−4.03	0.003	1.54	0.36	1.75	0.072
Zone 7	0.93	0.21	0.80	0.14	−13.27	0.000	0.84	0.14	−8.88	0.001	0.89	0.16	−4.46	0.048	0.90	0.17	−3.05	0.155
Total	9.46	1.35	8.79	1.28	−7.08	0.000	9.09	1.33	−3.85	0.000	9.41	1.38	−0.52	0.378	9.69	1.35	2.51	0.000

In ROI 1, highly significant bone loss (−14%) was observed in the first three months after surgery (*p* < 0.001). Despite a partial restoration of BMD after two years, the final value (−3%) was less than the initial value, which still represented a significant change (*p* < 0.05).

In ROI 2, an initial, highly significant reduction in the BMD value occurred (−9%), followed by a progressive increase in the BMD to the level of the baseline value, which was not significant in the first year (2%), but was significant after two years (5%), compared to the baseline value (*p* < 0.05).

In ROI 3, the BMD initially decreased (−2%) in the first three months; however, this loss was regained six months after surgery. The final BMD after two years (7%) resulted in a highly significant difference from the baseline value.

In ROI 4, an initial loss of BMD occurred, but it was regained after two years. A slight, gradual increase was noted between 12 months and 24 months. The final BMD was less than 1% greater than the baseline value.

In ROI 5, a highly significant initial loss of BMD occurred (−5%), followed by a constant increase in BMD until it was regained one year post-operatively, and at two years, the BMD gain was statistically significant (4%).

In ROI 6, BMD loss occurred (−9%) during the first three months (*p* < 0.001), but it was partially regained at 12 months. The BMD gradually increased from 12 to 24 months to reach 2% above the initial value, which does not represent a significant difference from the baseline value.

In ROI 7, BMD loss (−13%) occurred during the first six months (*p* < 0.001); gradual restoration was observed, but it did not reach the initial value after two years (−3%, not significant).

**Figure 3.**
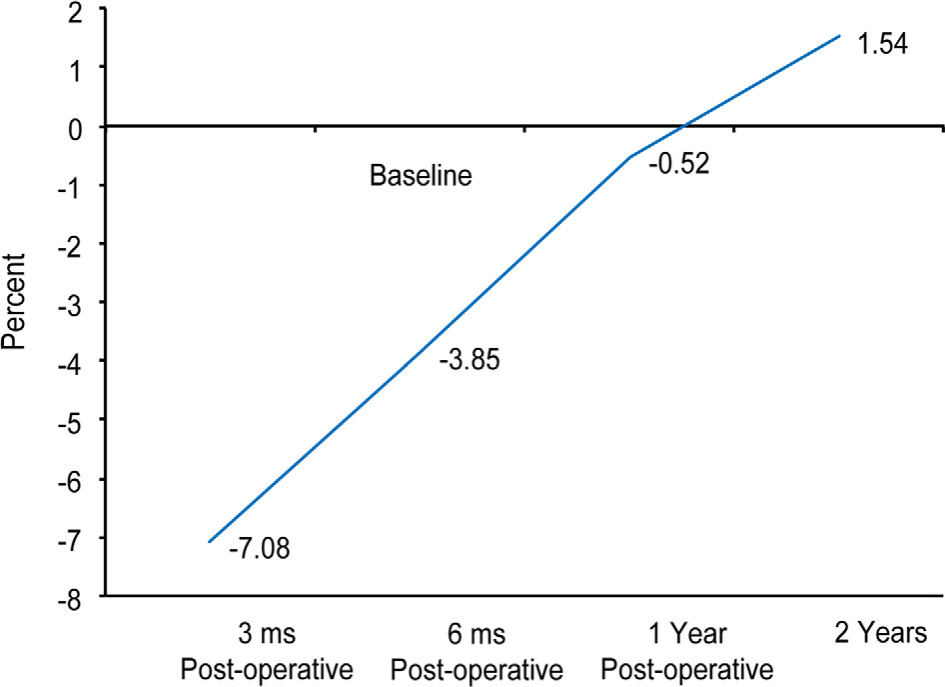
Graph showing the net changes in the BMD over a span of 2 years.

The **net mean** BMD decreased significantly during the first three months (−7%; *p* < 0.001) but returned to the initial value by one year. Thereafter, additional changes were observed. The final BMD after two years was almost 3% higher than the immediate post-surgical value making a statistically significant difference (Figure 3).

## Discussion

Short stem hip implants have been classified by Van Oldenrijk et al. into three categories: collum, partial collum, and trochanter-sparing stem designs. From the clinical point of view, they found unsatisfactory survival rate and accordingly, did not support the use of the collum designs. However, they reported promising medium-term results with the other two designs [3]. From the radiological point of view, few reports studied the bone remodeling process and changes in bone densitometry around the different short stem designs [10–13].

Concerning the partial column (neck preserving) designs, Lerch et al, in 2012, using the Metha short stem (B. Braun Aesculap, Tuttlingen, Germany) and the DEXA analysis, studied the changes in the bone densitometry after implantation in 25 patients. They found that BMD in the greater trochanteric region decreased significantly two years post-operatively. Minor changes were seen in the distal regions while in the lesser trochanteric region, significant increase was reported by 12.9% at two years follow-up. In the calcar region, BMD exceeded the baseline value by 6.1%. They concluded that the DEXA analysis showed load concentration on the medial part of the proximal femur and considered this an important guarantee for successful long-term results [10]. Similarly, at one year follow-up, Jahnke et al, in 2014, using the same implant in 40 patients, found a decreased bone density in ROIs 1, 4, and 7 and increased density in ROIs 2, 3, 5, and 6. They reported a hypertrophic bone remodeling process primarily in the lesser trochanter region by 6% [11].

A more proximal stress shielding was reported when studying the trochanter-sparing stem designs. In a retrospective BMD analysis following implantation of the Mayo (Zimmer, Winterthur, Switzerland) short stem, a significant decrease in ROIs 1, 6, and 7 was observed (ranging 14.4–17.9%), while much less affection was observed in ROIs 2–5 [12]. They concluded that there had been distal bony ingrowth with proximal resorption. Recently, Freitag et al when comparing the Fitmore short stem with CLS cementless straight stem (both Zimmer, Winterthur, Switzerland) noted also a stress protection of the proximal femur following trochanter-sparing short stem implantation. However, this stress protection was much less when compared with conventional stem type [13].

Using specific selection criterion, this prospective study was carried out to examine the bone behavior following the implantation of partial collum design short stem (MiniHipTM, Corin, Cirencester, UK). The MiniHip stem depends on metaphyseal ‘fit without fill’ for maximum bone conservation. Its medial curve follows the curve of the medial calcar for physiological load transfer. It is made of a titanium alloy with a hydroxyapatite coating on a titanium plasma spray in the proximal area of the stem.

DEXA is believed to be an efficient method for the evaluation of bone remodeling after hip arthroplasty. Several cross-sectional [14, 15] and longitudinal [16, 17] DEXA studies have been conducted after cemented and uncemented prothesis. In the present study, we used a longitudinal DEXA study protocol to assess bone remodeling. The initial BMD value obtained One week post-operatively was recorded as the baseline value to estimate the changes in the periprosthetic BMD in subsequent follow-up scans.

After surgery, the BMD in the overall periprosthetic area showed a significant reduction during the first three months. The most astounding reduction was recorded in the most proximal zones (ROIs 1 and 7), while the smallest reduction was observed in Zone 3, where the lateral part of the implant pushes against the lateral cortex. However, at 12 months, all the zones have regained some of their lost bone densities. In Zones 2, 3, 4, and 5 the BMD had reached a plateau near the initial values recorded shortly after the index procedure, with the highest increment recorded in Zone 3 laterally (mean + 3.3%). Statistically significant negative values were still recorded in the most proximal Zones 1, 6, and 7 with an average bone loss of 4–5% of the baseline values. When the two year BMD value after surgery was compared with the baseline value, two zones exhibited significantly lower BMDs (Zones 1 and 7), two zones had significantly higher BMDs (Zones 2 and 6), the change in BMD was highly significant in one zone (Zone 3), and two zones had non-significant increases in BMD (Zones 4 and 5). The net mean periprosthetic BMD was not statistically significant at one year but turned to be have a significant increase (+3%) at two years follow-up.

Our results were consistent with the previous records in the literature, denoting a process of bone resorption immediately after the surgery in all the periprosthetic areas. This was reversed, after the first three months post-operatively, to a process of bone formation that continues till two years. Significant changes occurred after the first year, especially in the lesser trochanteric region (Zone 6), which turned from a negative to a positive value after the first year. These changes did not support our hypothesis, present in the current literature [10–13], that only minor changes occur after the first year.

**Figure 4.**
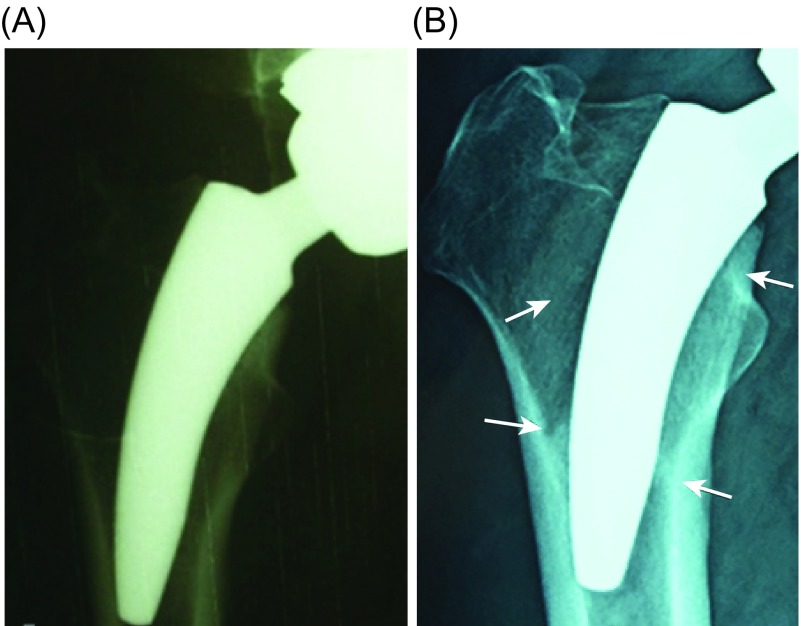
Anteroposterior radiographs of 40-year-old male: (A) immediate post-operative and (B) two years follow-up. The stem is considered in neutral alignment. Two years later; osseointegration evidenced by increased trabecular density bridging from lateral and medial cortex to the stem (spot welds – lower arrows) as well as rounding of the lower neck (upper arrows).

Bone resorption detected in Zone 7 (the lower neck) as well as the rounding off phenomena detected in 70% of the cases using the plain radiographs (Figure 4) over a span of two years follow-up, indicated stress shielding of this area. Contrary to this, increased BMD in the lesser trochanteric area indicated load concentration in the calcar area. This finding supports our hypothesis that neck preservation is an important factor for bone preservation in the calcar and the lesser trochanteric regions.

A remarkable observation in the current study is that when osseointegration between the bone and prosthesis is achieved, restoration of the periprosthetic bone is achieved.

Unification of the surgical procedure, using the DEXA, and careful patient’s selection are considered the main strengths of our study. However, the short follow-up period as well as the limited number of patients are considered the main weakness points.

In conclusion, the neck preserving MiniHip short stem implant has proven to be a bone-friendly design. Although bone resorption in the greater trochanteric region is still a problem, however, it has proven that the BMD in all the other periprosthetic regions including the calcar and the lesser trochanteric regions, are subjected to bone formation process over a period of two years.

## Conflict of interest

The authors declare no conflict of interest in relation with this paper.
